# The complete mitochondrial genome of *Anthus hodgsoni* (Passeriformes: Motacillidae)

**DOI:** 10.1080/23802359.2016.1192507

**Published:** 2016-07-23

**Authors:** Ping Sun, Chenling Zhang, Mujia Pang, Lifu Qian, Tao Pan, Hui Wang, Baowei Zhang

**Affiliations:** a School of Life Sciences, Anhui University, Hefei, Anhui, China;; b Faculty of Life Science and Chemical Engineering, Jiangsu Second Normal University, Nanjing, Jiangsu, China

**Keywords:** *Anthus hodgsoni*, mitochondrial genome, phylogenetic tree

## Abstract

*Anthus hodgsoni* is a species of small passerine bird in the family Motacillidae, which is widely distributed. In this study, we determined the complete mitochondrial genome of *A. hodgsoni*. The result showed that the total length of the mitochondrial genome was 16,886 bp and contained 2 ribosomal RNA genes, 22 transfer RNA genes, 13 protein-coding genes and 1 control region. The phylogenetic tree was reconstructed using the Bayesian analysis method and divided into four genera, *Anthus*, *Dendronanthus*, *Motacilla* and *Tmetothylacus*. The *A. hodgsoni* which we determined was clustered into genus *Anthus* and received strong support.

The olive-backed pipit (*Anthus hodgsoni*), classified as Least Concerned (LC) in the International Union for the Conservation (IUCN) Red List, is a small passerine bird of the pipit (*Anthus*) genus, which breeds across South, North Central and East Asia, as well as in the northeast of European Russia. They forage singly or in pairs on meadows and mainly feed on insects but also take on grass and weed seeds (MacKinnon et al. 2000). In the present study, the *A. hodgsoni* sample was collected from Huaining, Anqing of Anhui province, China (N 30°43′22.35″, E 116°49′27.68″). Now the specimen was deposited in the Laboratory of Evolution and Ecology, School of Life Sciences, Anhui University. Mitochondrial genome sequences have been proven to be useful for phylogenetic relationships at several taxonomic levels (Kumazawa & Endo 2004). We expect our result to provide a useful database for further research on *A. hodgsoni*.

The total genomic DNA of *A. hodgsoni* was extracted from muscle tissue using the standard proteinase-K/phenol–chloroform protocol (Sambrook & Russell 2006). Complete mitochondrial genome was amplified by polymerase chain reaction (PCR) using 16 pairs of primers then submitted it to GenBank (accession KX189345). The complete mitochondrial genome sequence of *A. hodgsoni* is 16, 886 bp in length and containing 13 protein-coding genes, 22 tRNA genes, 2 rRNA genes and 1 control region. The *ND6* subunit gene and eight tRNA genes (*tRNA^Pro^*, *tRNA^Gln,^ tRNA^Ala^*, *tRNA^Asn^*, *tRNA^Cys^*, *tRNA^Tyr^*, *tRNA^Ser^* and *tRNA^Glu^*) were encoded on the L-strand, the remaining genes were encoded on the H-strand. The gene arrangement is similar to the complete mitochondrial genomes of other Fringillidae species (Lerner et al. 2011).

Phylogenetic relationship of 35 Motacillidae species were analyzed with the Bayesian inference (BI) method using the MrBayes version 3.1.2 software (Ronquist & Huelsenbeck 2003) based on nucleotide sequences of *ND2* and *cytb* gene, selecting *Hemignathus flavus* as an outgroup. In this process, the best-fitting nucleotide substitution model (GTR + I + G) was selected via MrModeltest version 2.1 (Nylander 2004); the four independent Markov chain runs for 1,000,000 metropolis-coupled Monte Carlo (MCMC) generations, sampling every 1000 generations. When the average standard deviation of split frequencies reached a value less than 0.01, the first 1000 trees were discarded as “burn-in” and the remaining trees were used to calculate the Bayesian posterior probabilities (Pan et al. 2015). From the Bayesian analysis, the phylogenetic tree is divided into four genera and these relationships received strong support, with the topology similar to other research (Figure 1). The first clade was genus *Anthus*, included 24 species, of which *A. hodgsoni*1 and *A. hodgsoni* were clustered into the same clade which indicate that the species of our study was *A. hodgsoni*. The *A. hodgsoni* appears to be very closely related to *Anthus trivialis*. However, *Tmetothylacus tenellus* was deeply nested within *Anthus* may infer that this species was belonged to *Anthus*. The second clade is genus *Motacilla* and genus *Dendronanthus* form the last clade, moreover they are the sister group to the genus *Anthus* and *Tmetothylacus* (Alström et al. 2015).

**Figure 1.**
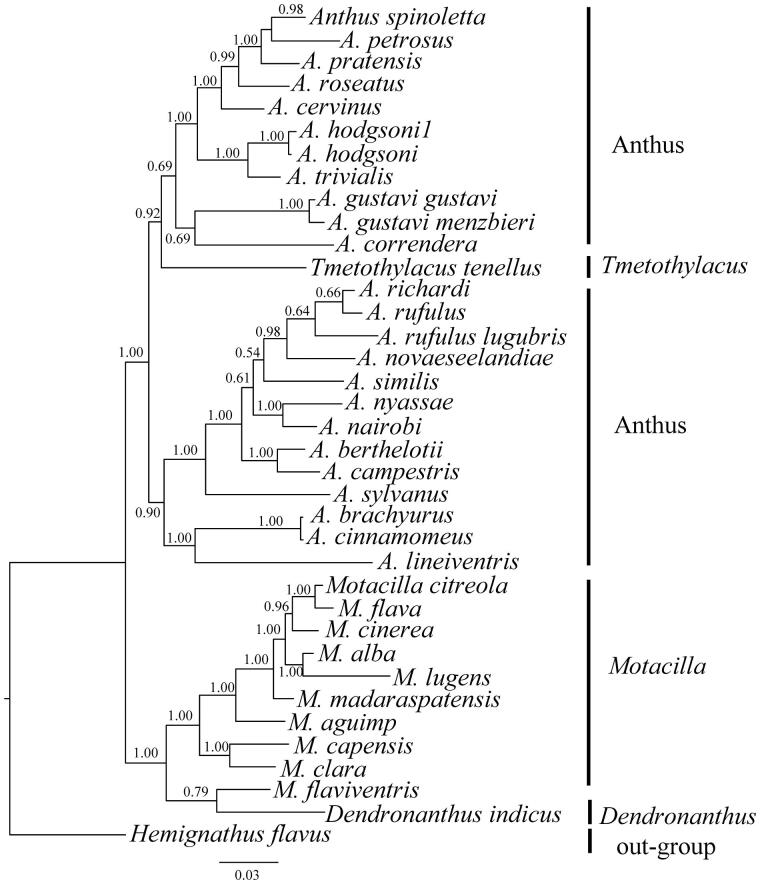
Inferred phylogenetic relationships among Motacillidae based on the ND2 and Cytb gene using Bayesian inference (BI). Numbers at each node indicate percentages of Bayesian posterior probabilities (BPPs). GenBank accession numbers for ND2 and Cytb are: *Anthus spinoletta *(None, LN650643), *A. petrosus* (None, APU46772), *A. pratensis *(AY259440, APU46774), *A. roseatus *(KJ455325, KJ456195), *A. cervinus* (None, ACU46776), *A. hodgsoni1* (KJ455323, KJ456193), *A. hodgsoni* (KX189345), *A. trivialis* (KP671568, None), *A. gustavi* *gustavi* (HM538381, None), *A. gustavi menzbieri* (HM538396, None), *A. correndera* (KP671555, None), *A. richardi* (None, ARU46770), *A. rufulus* (None, KJ456194), *A. rufulus lugubris* (KJ455324, None), *A. novaeseelandiae* (KC545397, KC545397), *A. similis* (KJ455326, J456196), *A. nyassae* (None, JQ796089), *A. Nairobi* (None, AM231754), *A. berthelotii* (None, EU047723), *A. campestris* (JN614730, JN614900), *A. Sylvanus* (KJ455327, KJ456197), *A. brachyurus* (None, AF526450), *A. cinnamomeus* (AY329410, AY329447), *A. lineiventris* (KP671561, None), *Motacilla citreola* (KJ455509, AF526442), *M. flava* (KC759314, AF526468), *M. cinerea* (KJ455508, NC027933), *M. alba* (NC029229, EU167005), *M. lugens* (NC029703, NC029703), *M. madaraspatensis* (KJ455510, KJ456348), *M. aguimp* (GQ369693, AF526466), *M.* *capensis* (AY329427, AY329464), *M. clara* (KP671575,AF526469), *M. flaviventris* (None, AF526446), *Tmetothylacus tenellus* (KP671579, None), *Dendronanthus indicus* (KP671571, None) and *Hemignathus flavus* (KM078780, KM078780).
